# Clinical spectrum of Posterior Reversible Encephalopathy Syndrome (PRES) in patients with eclampsia

**DOI:** 10.12669/pjms.315.7707

**Published:** 2015

**Authors:** Nazli Hossain, Nazeer Khan, Neelum Panhwar, Soobia Noureen

**Affiliations:** 1 Prof. Nazli Hossain, FCPS. Professor, Department of Obstetrics & Gynecology Unit-III, Dow University of Health Sciences, Karachi, Pakistan; 2 Prof. Dr. Nazeer Khan, PhD. Professor of Biostatistics, Jinnah Sindh Medical University, Karachi, Pakistan; 3 Dr. Neelum Panhwar, MBBS. Department of Obstetrics & Gynecology Unit-III, Dow University of Health Sciences, Karachi, Pakistan; 4 Dr. Soobia Noureen, MBBS. Resident Medical Officer, Department of Obstetrics & Gynecology Unit-III, Dow University of Health Sciences, Karachi, Pakistan

**Keywords:** Eclampsia, Posterior reversible encephalopathy syndrome, Pakistan

## Abstract

**Objective:**

To identify the prevalence, demographic details and clinical features of PRES in women suffering from eclampsia.

**Methods:**

Women admitted in the labor room suite with diagnosis of eclampsia were studied. The study period was from October 15. 2011 to March 15. 2012, in the department of obstetrics & gynecology unit 3, Civil hospital Karachi. Of all patients with diagnosis of eclampsia, 22 underwent neuro imaging by computerized tomography. Demographic details, clinical findings and maternal and perinatal outcome were entered on a predesigned Performa.

**Results:**

Thirty four women were identified, with eclampsia. Neuro imaging was done in 22 women. Posterior reversible encephalopathy syndrome was recognized in 9 (22) patients. The mean systolic blood pressure was 161(±11) mm Hg, and mean diastolic blood pressure was 111(±10) mm Hg. Mean number of fits were three, and the mean gestational age of patients were 35 weeks. Gestational age was found significantly associated with PRES (p <0.3) Mean leukocyte count in patients with eclampsia was 20,083±16,165/cu mm.

**Conclusion:**

Our study shows presence of Posterior reversible encephalopathy syndrome (PRES) in women who are identified with eclampsia. There is need for awareness and long term neurological follow up in this group of women.

## INTRODUCTION

Hypertensive disorders of pregnancy include pregnancy induced hypertension (PIH), pre eclampsia (PE) and eclampsia. In developing countries, eclampsia is responsible for 31% of maternal deaths.^1^ In one of our own study, conducted at Civil Hospital Karachi hypertensive disorders were responsible for 15% of maternal deaths.^2^ Pre eclampsia (PE) is defined as new onset hypertension (systolic ≥ 140mm Hg, and diastolic blood pressure of ≥90mm Hg) after 20 weeks of gestation, along with proteinuria (> 300mg/dl) in 24 hours. Severe Pre eclampsia (sPE) is defined as systolic pressure ≥ 160 mm Hg and diastolic blood pressure of ≥ 110mm Hg with proteinuria of 2gm/dl, along with clinical features of severe headache, blurring of vision, epigastric pain and oligura. Eclampsia is defined as generalized convulsions in a woman with sPE in absence of any other cause.^3^ Eclampsia is associated with cardiovascular and neurological complications. Neurological complications include visual disturbances, cerebral edema, posterior reversible encephalopathy syndrome (PRES) and intracranial hemorrhage. Later is main cause of fatality in eclampsia.

Posterior reversible encephalopathy syndrome (PRES) is a neuro-radiological syndrome, characterized clinically by altered consciousness, seizures and visual disturbances. PRES is seen in eclampsia, due to acute cerebral injury. Neuro imaging by computed tomography (CT) and magnetic resonance imaging (MRI) helps in reaching the diagnosis. Neuroimaging shows presence of bilateral and symmetric brain edema, typically seen as hypodense areasmainly in subcortical regions of occipital and parietal lobes. It is important to recognize this condition, as control of blood pressure, helps in early reversal of symptoms, and minimizes long term sequale.

Our study aimed to look at the prevalence, clinical features and neuro-radiological findings of PRES in women diagnosed with eclampsia.

## METHODS

This study was done at the department of obstetrics & gynecology Unit-3, Civil Hospital Karachi and Dow University of Health Sciences. The study period was from 15 October 2011 to 15 March 2012. During this period, a total of 34 women were admitted in the labor room suite with diagnosis of eclampsia. It was defined as presence of tonic-clonic convulsion in women with severe PE. Patients are managed by a multidisciplinary team, which includes senior obstetrician, intensivisit and neurologist. Computerized tomographgy (CT) and Magnetic resonance imaging (MRI) are requested by neurologist. Due to cost constraint, CT scans are advised first, followed by MRI, if required to reach a conclusive diagnosis. Facilities for CT scan are available free of cost at the hospital. Magnesium sulphate is given as standard loading dose, followed by the maintenance dose. These patients underwent computed tomography (CT), at least 2 days after the last seizure activity. The CT report was evaluated by the radiologist, individually. Neurologic abnormalities were noted.

Demographic and clinical details were noted on a predesigned Performa. Women were divided in two groups, A included women with findings of PRES, and B included eclamptic women with out findings of PRES on computed tomography. Data was entered on SPSS Version 16. Statistical analysis was made using Mann Whitney test.

## RESULTS

During the study period from 15 October 2011 to 15 March 2012, a total of 1440 deliveries were conducted in the department. Diagnosis of eclampsia was made in 34 women, hence giving a prevalence of 2.36%. Computed tomography (CT) results were available in 22patients. A total of nine women were identified with PRES. The women were divided in 2 groups, Group A, with findings of PRES, and Group B, without PRES. After clinical stabilization of blood pressure, these women underwent computed tomography of brain. In all women in whom a diagnosis of PRES was made, occipital lobe showed presence of infarction, edema, or hemorrhage. Mean age of patients was 24.73± 6.9 years. The mean systolic blood pressure for those in whom diagnosis of PRES was made was below 180mmhg (161 mm hg). Mean number of fits were three, and the mean gestational age of patients were 35 weeks. Mean leukocyte count in patients with eclampsia was 20,083±16,165/cu mm, and mean serum creatinine was 0.9±0.3 mg/dl. Mean number of hospital stay was 5±1 days.

We compared clinical and demographic features in women diagnosed with and without PRES.(Table-I) We did not find any statistical difference between blood pressure and number of fits in both groups. Gestational age was significantly associated with presence of PRES (p <0.03) Cortical blindness was seen in one patient, in each group, followed by complete recovery.

**Table-I:** Clinical and demographic comparison of eclamptic women with and without PRES diagnosis.

	Group A (n=9) PRES	Group B (n=13) WITHOUT PRES	P value
Age (years)	26.2±8.3	23.7±6.3	0.435
Parity	1.67±3.2	0.92±1.8	0.845
Gestational age(weeks)	34.3±3.2	36.5±2.8	0.030
Systolic blood pressure (mmHg)	161.1±14.5	163.1±10.3	0.845
Diastolic blood pressure (mmHg)	111.1±11.7	113.9±10.4	0.744
Number of fits	2.8±1.1	3.8±5.1	0.262

## DISCUSSION

Our study in eclamptic women, show strong evidence for presence of neuro radiological evidence of PRES. It was seen in 9(22), forty percent of women, diagnosed with eclampsia. Table-I shows the clinical and demographic data of women with eclampsia. We found a raised white blood cell count in women with eclampsia, suggesting an inflammatory etiology for eclampsia. This has been observed by other investigators as well.^4^,^5^ Majority of the women were primipara, except 4 women who were multiparous. Headache was seen in all patients, whereas blurring of vision was seen in 6(22 These are women with eclampsia) patients.

We found PRES, affecting mainly the occipital lobe of brain. Findings included presence of hypo dense areas on CT scan, along with radiological evidence of edema. The degree of brain edema found on imaging has been linked to severity of PRES.^6^ This is in aggrement with other studies, where posterior part of brain has been found to be more affected. This has been attributed to easy break down of blood-brain barrier, and decreased ability to respond to increased blood pressure.^7^ In the initial observation by Hinchey J, also posterior part of brain was found more affected, as compared to other parts of brain.^8^ This finding has recently been challenged. In a small series, investigators have found brain lesions affecting other parts of brain, compared to posterior region.^9^ Both grey and white matter are affected, hence the term reversible leuckoencephalopathy has been termed as misnomer.^10^

We also found women in group A, having less number of eclamptic fits, compared to women in Group B, though it was not significant. (Table-I) Both groups had multiple seizures. PRES due to eclampsia has been found to be associated with multiple seizures.^11^ The mean gestational age for eclamptic fits was 35 weeks in our study. PRES has also been reported in at term woman and in a woman at 27 weeks of pregnancy.^11^,^12^ Significant association of PRES was seen with gestational age. PRES has also been observed in post partum period.^13^ In our study women who had PRES, were older, compared to women who did not have PRES on imaging (26 years vs 23 years) In a study by Wagner SJ, eclamptic women with PRES, were found to be younger, mean age 20 years.^14^ Due to small sample size we can not comment on greater maternal age in our study population.

The blindness which is seen in above group of women is termed as cortical blindness, as pupillary reflex, normal ocular movements are not affected in PRES. Vision is usually reverted back to normal with in 2-48 hrs. We had one patient in each group, who suffered from blindness, due to extreme blood pressure. In both cases vision was restored completely in 24 hours.

Ours is a public sector hospital, which receives referrals from a vast area. Pakistan has a high maternal mortality due to eclampsia. It is necessary that local obstetricians should be aware of this diagnosis, in patients with eclampsia.

It is important to recognize PRES in women with eclampsia. Prompt and aggressive control of blood pressure is the cornerstone of the management. This results in early resolution of neurological changes seen on MRI. Delay in diagnosis and treatment may result in cerebral ischemia and infarction.^11^ Delay in diagnosis of PRES has also been reported to be associated with permanent neurological deficit and death.^4^ If not recognized, it may lead to either hemorrhage or infarction.^10^ Long term follow up is equally important in above group of women.
